# Outer packaging labelling of medicines in Southern African Development Community (SADC) countries: comparative analysis of requirements and transition terms for harmonisation

**DOI:** 10.1186/s12913-024-10585-0

**Published:** 2024-01-20

**Authors:** K. Narsai, F. B. Masekela, H. G.M Leufkens, A. K. Mantel-Teeuwisse

**Affiliations:** 1 Division of Pharmacoepidemiology & Clinical Pharmacology, Utrecht Institute for Pharmaceutical Sciences (UIPS), Utrecht University, Utrecht, The Netherlands; 2 Nelson Mandela School of Public Governance, Cape Town, South Africa; 3 Medicines Control Authority of Zimbabwe (MCAZ), Harare, Zimbabwe

**Keywords:** Regulatory harmonisation, Labelling, Medicines, Africa, Pharmaceutical, SADC, Outer packaging

## Abstract

**Introduction:**

The COVID-19 pandemic highlighted an urgent need for harmonised requirements for the regulation of medicines. To fully implement harmonised medicines regulations across Africa, common technical standards of medicine regulations are needed. One such technical standard is the labelling of medicines on outer packaging. In this study, we compared outer packaging labelling requirements and transition terms for harmonization for countries in the Southern African Development Community (SADC) region.

**Methods:**

Data on legislation and/or regulatory guidelines for medicine outer packaging labelling from National Medicines Regulatory Authorities (NMRAs) were obtained for countries in the SADC region (*n* = 16) by February 2023. A detailed comparative content analysis was conducted to determine alignment with the requirements of the Southern African Development Community (SADC) harmonised labelling guidelines to assess readiness levels of each country to transition to the SADC harmonised labelling guideline for outer packaging of medicines.

**Results:**

Content analysis showed at least 11 out of 16 countries require national legal reform to transition to the SADC harmonised labelling guideline. In all cases where countries specified labelling requirements for outer packaging of medicines, these were stipulated in national medicines legislation.

**Conclusion:**

Even though there is a high level of alignment across the countries in terms of national labelling requirements, most countries in the SADC region would still require national legislative reform to transition to regional harmonised labelling requirements and then ultimately to continental requirements of the African Medicines Agency (AMA).

## Introduction

Effective regulation of medicines and vaccines is vital for the protection of public health by ensuring adherence to predetermined standards of quality, safety and efficacy. To make this vision of regulatory harmonisation a reality, various regional regulatory harmonisation initiatives were implemented across the continent [1]. Significant progress has been made in implementing collaborative review procedures to circumvent the resource constraints of National Medicines Regulatory Authorities (NMRAs) through the African Medicines Regulatory Harmonisation Initiative (AMRH) and regional joint assessment procedures using reliance models [1–5]. The lack of resources and differences in the time of implementation of recommendations by individual countries have been identified as challenges to the implementation of regulatory harmonisation [6]. 

The ZAZIBONA program was launched within the Southern African Development Community (SADC) region, in 2013 sought to streamline regulatory review processes through joint assessment procedures and making recommendations for medicines registration, which remains a national process [6–8]. To leverage opportunities such as work sharing; it is important to have common technical standards across SADC countries. The alignment of national regulations and standards with the regional harmonised standards will be critical to successful implementation of regulatory harmonisation for medicines, first at a regional economic community (REC) level and then at a continental level through the AMA [1]. 

A typical example of such technical standards relates to the labelling of medicines on the outer packaging, which not only allows regulators to assess the authenticity of medicines but also patients to correctly identify medicines. to avoid medication errors [9–11]. Even though various definitions for ‘label’ or ‘labelling’ can be found both in legislation as well as in published literature, common elements include the following: any brand, written, pictorial or descriptive matter appearing on or attached to a package of a medicine [1, 6, 8, 12–16]. 

Regulatory requirements for labelling of medicines which companies need to adhere to are either included in national medicines legislation or detailed in technical guidelines [1, 17]. These labelling requirements include information appearing on the outer packaging, immediate packaging and information leaflet contained inside the medicine pack. Labelling requirements for outer packaging of medicines include manufacturing details, distribution details and country specific registration numbers indicating successful registration by an NMRA.

Evidence from several studies that investigated levels of compliance to labelling requirements demonstrated that marketed medicines showed varying levels of compliance, including failure to comply, such as the example from the East Africa Community (EAC), which showed varying levels of compliance even in the presence of harmonised medicine regulations [13, 16, 18]. This is further emphasized by Barton, et al. who cite redundant and non-transparent registration procedures which are widely divergent across countries, including complex country specific labelling requirements and GMP inspections, as problematic for companies to implement. Such complex regulations are reasons why pharmaceutical companies may decide against supplying medicines to specific markets altogether with the resultant impact of higher medicine costs, limited market competition, limited medicine choices which in turn exacerbate public health and social challenges [1, 13, 14, 19]. 

The SADC guideline on labelling of medicines on outer packaging, titled “Guideline on excipients in the labelling, summary of product characteristics and patient information leaflet of medicinal products for human use “, (hereafter referred to as the SADC Labelling Guideline) seeks to harmonise these requirements across the SADC region and proposes additional requirements which are not currently implemented at SADC country level such as QR coding [20–22]. It is hypothesized that the benefits of harmonisation can be achieved at each country level without the need for national legal reform of medicines legislation but rather through implementation of common technical standards for labelling of medicines. This hypothesis was tested using comparative content analysis of medicine labelling requirements in national medicines legislation and technical guidelines in force in SADC member countries [22]. 

## Methods

This study aimed to assess to what degree requirements for the labelling of medicines on outer packaging in all SADC countries, contained in national medicine legislation and technical guidelines, are consistent with the SADC Labelling Guideline through a comparative content analysis of legislation and technical guidelines governing the requirements for labelling of medicines at the national level in SADC countries. The level of change that will be required for individual SADC countries to transition from country specific labelling requirements to implementing the harmonised SADC guidelines was determined.

The 16 countries in the SADC region of Africa are: Angola, Botswana, Comoros, Democratic Republic of Congo, Lesotho, Madagascar, Malawi, Mauritius, Mozambique, Namibia, Seychelles, South Africa, eSwatini, Tanzania, Zambia and Zimbabwe. These countries differ in terms of levels of development, market sizes, income levels and languages (French, Lusophone and Anglophone) and infrastructure [23]. 

### Data collection

**Fig. 1:**
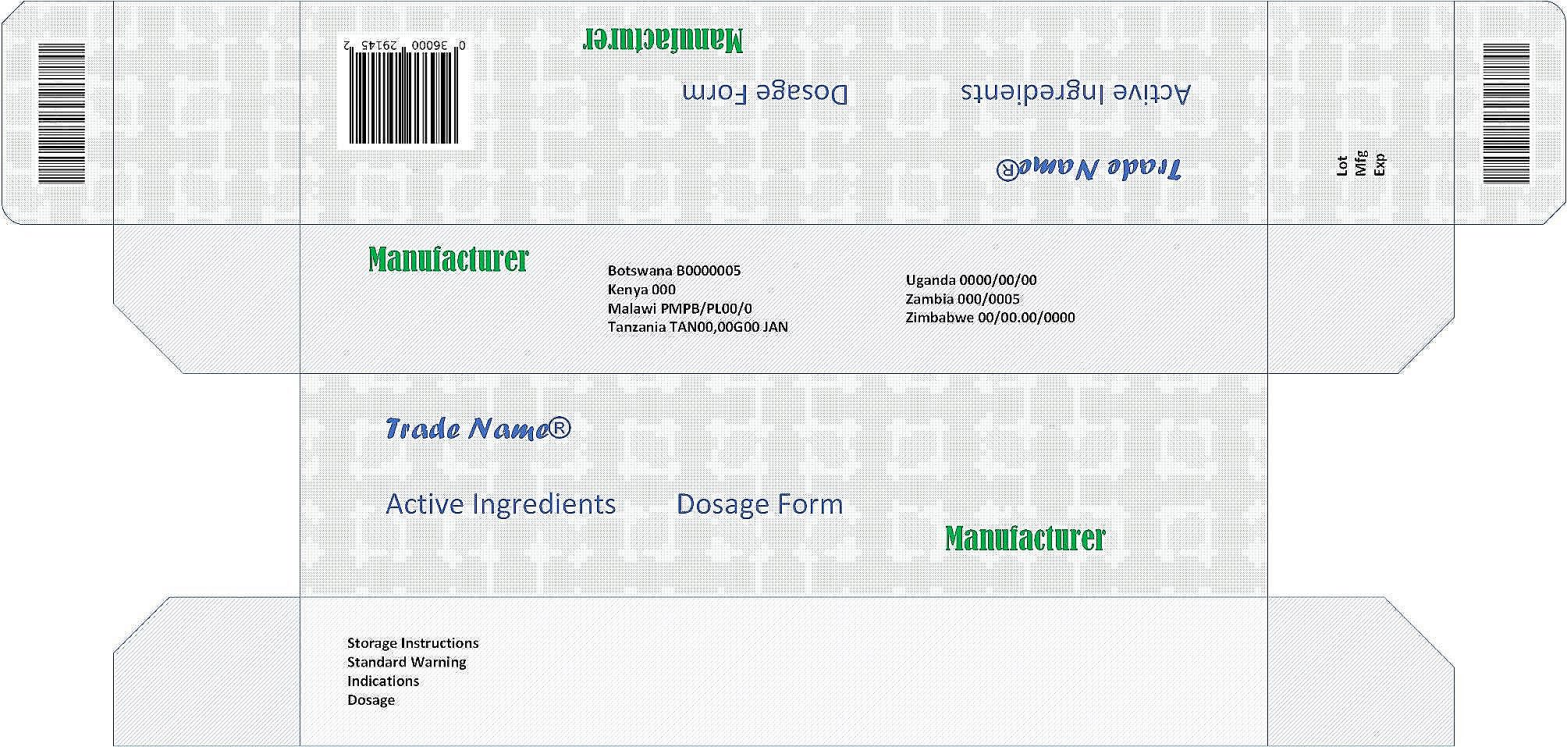
Medicine pack illustration with labelling illustration

For the purposes of this study, medicine labelling requirements were limited to the information printed on the outer packaging of medicines, (Fig. 1), as required by NMRAs in the SADC region, as part of requirements for medicines registration.

Extensive internet research was conducted to obtain national legislation and/or technical guidelines during February 2023 using multiple search strategies, including NMRA websites, WHO online resources, published literature, pharmaceutical trade association and professional association resources to determine the status of country specific labelling requirements. In addition, to augment the online research and compensate for data gaps, direct engagement with national academics, policymakers and regulators within the researchers’ network was conducted for data verification.

### Data analysis

The first step was to determine whether SADC countries have a functioning NMRA and implemented medicines legislation and legal requirements for medicines registration. Legislation was reviewed to determine whether the labelling requirements were included in principal medicines legislation or in technical guidelines.

The SADC labelling guideline was reviewed to identify the specific labelling attributes included [21]. The following attributes were identified: trade name, dosage form, packaging unit size, manufacturer name, batch number, manufacturing date, expiry date, manufacturers’ details, storage information, standard warning, QR code and lastly registration number and scheduling status. Regulatory requirements for outer packaging labelling were reviewed for each SADC country including any country specific requirements, such as scheduling status and registration numbers. These requirements were compared to the SADC guideline, including, to determine how SADC countries would transition to adopting the SADC harmonised guideline.

## Results

**Fig. 2:**
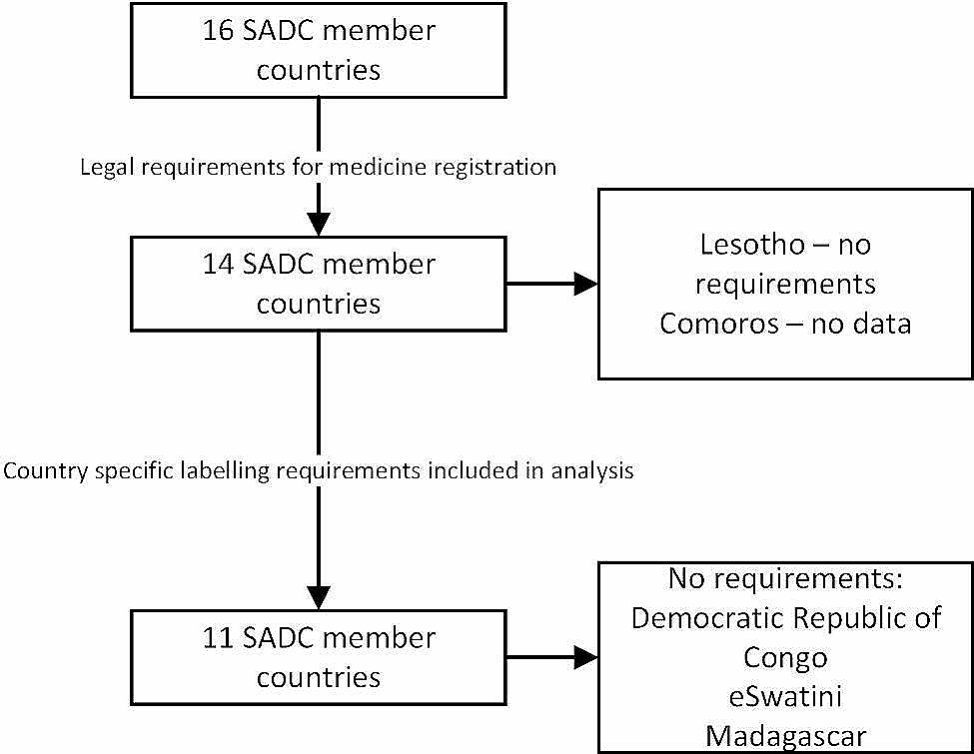
High level analysis of SADC countries labelling requirements

Our analysis showed that all SADC countries have a NMRA and that 14 out of 16 countries have legal requirements for the registration of medicines with the exception of Lesotho. No data was found for Comoros. These two countries were excluded from further analysis (Fig. 2).

Country specific requirements for outer packaging labelling were found in 11 out of 16 SADC countries (69%). Three countries do not require country specific labelling, viz., Democratic Republic of Congo, eSwatini and Madagascar. These countries accept outer packaging labelling from other countries, even though this is not explicitly stated in their respective national regulations.

In all cases where SADC countries specified labelling requirements, (*n* = 11/16), these were stipulated in national medicines legislation, e.g., the Medicines Acts governing the regulation of medicines and not in technical guidelines. Further detailed analysis of the specific attributes of medicine labelling required on the outer packaging was limited to these 11 countries that were found to have requirements for country specific labelling.

There was consistency in outer packaging labelling attributes across these 11 countries with the exception of Mozambique and Zambia. For these countries deviations were noted in the requirements for the following attributes: recommended dosage, manufacturers name, batch number, manufacturing date and manufacturers details. Madagascar does not require packaging unit size. South Africa requires manufacturers name, batch number, expiry date while manufacturing date and manufacturers details were optional. This is similar to the requirements for Zambia (Table 1). It was further observed that no countries had requirements for QR code printing. However, this is a requirement in the SADC labelling guideline [21, 24–51]. 

**Table 1:** Outer packaging labelling requirements for countries with country specific labelling legislation [24–51]

Country	Active Ingredient	Trade Name	Dosage Form	Packaging Unit Size	Dosage	Manufact Name	Batch Number	Manufact Date	Expiry Date	Manufact Details	Reg Number	Storage Info	Standard Warning	Scheduling Status	QR Code
SADC Guideline	Y	Y	Y	Y	Y	Y	Y	Y	Y	Y	Y	Y	Y	Y	Y
1. Angola *	Y	Y	Y	Y	Y	Y	Y	Y	Y	Y	Y	Y	Y	Y	**N**
2. **Botswana**	Y	Y	Y	Y	Y	Y	Y	Y	Y	Y	Y	Y	Y	Y	**N**
3. **Malawi**	Y	Y	Y	Y	Y	Y	Y	Y	Y	Y	Y	Y	Y	Y	**N**
4. **Mauritius**	Y	Y	Y	Y	**N**	**N**	**N**	**N**	Y	**N**	**N**	Y	Y	N	**N**
5. Mozambique*	Y	Y	Y	Y	**N**	**N**	**N**	**N**	Y	**N**	**N**	Y	Y	Y	**N**
6. **Namibia**	Y	Y	Y	Y	Y	**N**	Y	**N**	Y	**N**	Y	Y	Y	Y	**N**
7. **Seychelles**	Y	Y	Y	Y	**N**	**N**	Y	Y	Y	Y	Y	Y	Y	Y	**N**
8. **South Africa**	Y	Y	Y	Y	Y	**N**	Y	**O**	Y	**O**	Y	Y	Y	Y	**N**
9. **Tanzania**	Y	Y	Y	Y	Y	Y	Y	**Y**	Y	Y	Y	Y	Y	Y	**N**
10. **Zambia**	Y	Y	Y	Y	Y	Y	Y	**N**	Y	**N**	Y	Y	Y	Y	**N**
11. **Zimbabwe**	Y	Y	Y	Y	Y	Y	Y	Y	Y	Y	Y	Y	Y	Y	**N**

Y = aligned with SADC guideline requirements

N = not aligned with SADC guideline requirements

O = optional requirement in national legislation

* specific language requirements

Some countries, Botswana, Namibia and Tanzania have requirements for labelling pertaining to specific active ingredients such as paracetamol and/or preservatives as well as non-pharmacological information including reimbursement and pricing information and volume specifications for specific formulation types such as eye drops and ointments (Table 2).

**Table 2:** Specific technical requirements in medicine legislation for countries which include requirements for outer packaging labelling [24–51].

Country	Medicines Legislation
Angola	None
Botswana	Information required for preservatives, ingredients such as paracetamol, aspirin and specific formulations, e.g., oral rehydration salts
Malawi	None
Mauritius	Requirement to include the following statement, “MOH & Wellness NOT FOR SALE”
Mozambique	None
Namibia	Information required for preservatives, ingredients such as paracetamol, tartrazine
South Africa	Requirements for barcode suitable for identification and tracking; South Africa is the only country that has provision for inclusion of regulatory information from other jurisdictions, i.e., manufacturer details and date, scheduling status and registration number allocated by other NMRAs
Seychelles	None
Tanzania	Special precautions for disposal of unused medicines or waste material from unused medicines, reimbursement conditions for social security, identification and authenticity, statement re government ownership of medicine
Zambia	None
Zimbabwe	Name and percentage of bacteriostatic or bactericidal agents

For Francophone and Lusophone countries, Mozambique and DRC, language requirements need to be accommodated. For example, Mozambique required that language requirements be adhered to in terms of information appearing in Portuguese on labelling. No data was found for Angola, Comoros and DRC.

## Discussion

The detailed analysis of labelling requirements showed that at least 11 out of 16 countries (69%) will require legal reform at a national level to align their labelling requirements to those of the SADC Labelling guideline final draft dated February 2020.

Labelling requirements stipulated in technical guidelines, i.e., not in medicines legislation, can be updated without the need for legal reform. None of the countries which specified labelling requirements did so in technical guidelines in this study. For the 4 countries which do not currently specify country specific labelling, minor reforms will be required to ensure that the SADC Labelling guideline is implemented and enforced at a national level. For the remaining 8 countries where country specific labelling requirements are contained in national medicines legislation, more significant legal reform will be required to amend this legislation to align with the SADC Labelling guideline. Even though joint assessment procedures have been implemented at a REC level, this does not circumvent the need to adhere to national legislation for medicines registration.

Most significantly, the SADC labelling guideline does not propose any harmonization of requirements for scheduling status and registration numbers which are country specific. Depending on the country’s policymaking processes, this may involve national parliamentary processes which can be lengthy,adding to the complexity of implementation [4]. Although treaties and protocols for medicines regulatory harmonisation exist, such as the AMA Treaty, implementation of agreed regional decisions by countries remains challenging, since treaties are not self-executing, hence requiring domestication of such decisions through national legal reform.

### Recommendations for implementation of harmonised labelling for outer packaging of medicines in the SADC region

It is clear from the results of this study that the majority of countries in the SADC region will need to undergo national legal reform of existing medicines legislation to accommodate and/or transition to the harmonised SADC guideline for labelling of medicines. However, there is currently no binding requirement for countries to transition to harmonised regulatory processes while national medicines legislation remains enforced. In parallel, at an African continent level, there has been an increased focus and momentum on the establishment of African Medicines Agency (AMA) which will further promote the development and adoption of harmonised regulatory policies and standards for the regulation of medical products across the African continent [52]. momentum should be leveraged to develop operational plans at a country level to implement harmonised medicine regulatory policies such as those related to labelling requirements for outer packaging of medicines.

### Limitations to the study

Information regarding labelling requirements for each of the countries was challenging to find through online resources as not all the NMRAs have comprehensive, up-to-date online resource portals or websites. In some cases, information was obtained through email contact with key policy officials, academics in-country and professional organisations of healthcare professionals such as pharmacists. In some cases, information provided could not be verified through relevant documentation.

Legislative information differed in phrasing and needed to be interpreted for purposes of standardisation for the analysis. Sigonda et al., claimed that all African countries have a functioning regulatory authority. In this study, we were not able to confirm this using the various methods described. Finally, legislation, such as that from Angola, is published in Portuguese and therefore needed to be translated since the authors are mainly English-speaking.

## Conclusion

The majority of SADC countries currently have country specific regulatory requirements for outer packaging labelling of medicines which are legislated The SADC Labelling Guideline aims to harmonise these labelling requirements across the SADC region. Further strengthening of the operationalisation of the AMA could assist in increasing the momentum of harmonisation by creating a pathway for countries to transition to harmonised regulatory requirements. Further research is recommended to investigate the impact on NMRAs and pharmaceutical companies in the implementation of harmonised regulatory requirements in the absence of binding legal frameworks at a regional and continental level.

## Data Availability

The datasets used and/or analysed during the current study are available from the corresponding author on reasonable request. All data generated or analysed during this study are included in this published article.
